# Microbe Profile: *Pseudomonas aeruginosa*: opportunistic pathogen and lab rat

**DOI:** 10.1099/mic.0.000860

**Published:** 2019-10-10

**Authors:** Stephen P. Diggle, Marvin Whiteley

**Affiliations:** ^1^​ Center for Microbial Dynamics & Infection, School of Biological Sciences, Georgia Institute of Technology, Atlanta, GA 30332, USA

**Keywords:** *Pseudomonas aeruginosa*, Genomics, Physiology

## Abstract

*
Pseudomonas aeruginosa* is a Gram-negative opportunistic pathogen and a model bacterium for studying virulence and bacterial social traits. While it can be isolated in low numbers from a wide variety of environments including soil and water, it can readily be found in almost any human/animal-impacted environment. It is a major cause of illness and death in humans with immunosuppressive and chronic conditions, and infections in these patients are difficult to treat due to a number of antibiotic resistance mechanisms and the organism’s propensity to form multicellular biofilms.

## Taxonomy

Kingdom Monera, phylum Proteobacteria, class gamma sub-division, order *Pseudomonadaceae *, genus *Pseudomonas *, species *Pseudomonas aeruginosa *.

It was the French pharmacist Carle Gessard who first described *P. aeruginosa* in his study ‘On the blue and green coloration of bandages’ in 1882. *P. aeruginosa* produces a number of pigments in culture, but it is likely that Gessard was describing pyocyanin, a blue/green phenazine compound that has both antimicrobial and toxin properties. The name *Pseudomonas* is derived from two Greek words: *Pseudo* meaning ‘false’ and *monas* meaning ‘single unit’; *aeruginosa* ‘greenish-blue’ is from the latin aerūgō meaning ‘rusted copper’.

## Properties

*P. aeruginosa* is a heterotrophic, motile, Gram-negative rod-shaped bacterium about 1–5 µm long and 0.5–1.0 µm wide. It is a facultative aerobe that grows via aerobic respiration and anaerobic respiration with nitrate as the terminal electron acceptor. *P. aeruginosa* can also grow anaerobically with arginine and has limited fermentative abilities that generally support very slow or no growth. The organism can utilize over 100 organic molecules as a source of carbon and/or energy and as a prototroph, generally has the ability to grow on a minimal salts growth medium with a single source of carbon and energy. *P. aeruginosa* grows well at 37 °C, but it can survive in broad temperatures ranging from 4–42 °C. It is an important soil bacterium that is capable of breaking down polycyclic aromatic hydrocarbons, but is often also detected in water-reservoirs polluted by animals and humans, such as sewage and sinks inside and outside of hospitals. The two most common laboratory strains used are PAO1 [1] and PA14, both of which have been used to create genomic resources including publicly available ordered transposon mutant libraries. In addition, a highly curated on-line resource is currently available at www.pseudomonas.com, which actively integrates new genomic and molecular findings, and PAMDB (http://pseudomonas.umaryland.edu) is a comprehensive *P. aeruginosa* metabolome database.

*P. aeruginosa* is often resistant to many classes of antibiotics and therapeutic agents, and this makes it problematic during infection as it can be difficult to treat. It is often termed an ‘opportunistic’ pathogen because it rarely infects healthy individuals. Clinically, the primary risk is for patients with compromised immune systems including those with cystic fibrosis (CF), cancer, AIDS, indwelling medical devices, burn and eye injuries, and non-healing diabetic wounds.

## Phylogeny

Initial studies using core genome SNP phylogeny subdivided *P. aeruginosa* strains into two major groups (group I, which includes strain PAO1, and group II, which includes strain PA14) and one minor group of mostly unrelated clonal lineages. More recently, pan-genome studies have revealed a five-group population structure of *P. aeruginosa* [2]. The two new groups are intermediate between groups I-II and group III, which is genetically distant from all other groups. Whether there is significant correlation between environmental and clinical strains remains to be determined.

## Genome and evolution

The first *P. aeruginosa* genome sequenced was that of strain PAO1 [1], originally a chronic wound isolate from the 1950s and now a commonly used laboratory strain. The sequence of PAO1 is 6.3 Mbp in size (5570 predicted open reading frames), which in the year 2000, made it one of the largest bacterial genomes sequenced. It is important to note that there is phenotypic and genomic variation between PAO1 strains stored in different laboratories throughout the world, which is a consideration for the reproducibility of experiments between different research groups. Another well-used laboratory strain is PA14, originally isolated from the environment as a highly virulent strain that caused extensive plant rot, but which has also been isolated from human burn wounds. The *P. aeruginosa* genome contains a large number of transcriptional regulators and many genes involved in catabolism, transport and efflux of organic compounds. This genomic and metabolic flexibility is likely to be key for allowing *P. aeruginosa* to colonize and thrive in a range of environments.

The genomic era has resulted in hundreds of *P. aeruginosa* genomes from a range of infection and non-infection environments. Recent bioinformatic analyses have revealed a core genome and a significant pan-genome [2]. A study using nine strains of *P. aeruginosa,* and a transposon insertion sequencing (Tn-Seq) approach to define essential genes for fitness on five different media, revealed 321 core essential genes, representing 6.6 % of the genome [3]. Another study used over 1300 *P *. *aeruginosa* genomes sourced from a variety of environments. Here, it was shown that the *P. aeruginosa* pan-genome consists of 54 272 genes: 665 of them core genes, 26 420 flexible genes and 27 187 unique genes [2]. Horizontal gene transfer is also likely to be important and there were over 3000 putative and fragmented plasmids containing resistance and virulence genes identified. Clinical isolates of *P. aeruginosa* often carry several large prophages (absent in PAO1, except for the Pf1-like filamentous phage Pf4), which may contribute to bacterial fitness and virulence and the lysis of competitor strains.

Antimicrobial resistance (AMR) is a significant problem in some clinical strains. AMR in *P. aeruginosa* can occur by (a) acquisition of resistance genes via horizontal gene transfer; or (b) mutations in genes already present in the genome, leading to up-regulation of efflux pumps, beta-lactamase’s or changes in porins. Carbapenemase-resistant *P. aeruginosa* strains are amongst the critical pathogens listed on the WHO priority pathogens' list.

## Key features and discoveries

*P. aeruginosa* is capable of causing disease in a variety of hosts including plants, nematodes, insects and mammals. In humans, it is particularly problematic in patients with CF. In CF lungs, infection often occurs early in life and despite aggressive treatment with antibiotics, infection results in a progressive loss of lung function and eventually death [4]. Interestingly, adaptation of *P. aeruginosa* during chronic infection, often leads to a loss of virulence determinants including motility, O-antigens, type III secretion and quorum sensing (QS). Using model systems, a large number of functions have been shown to be important for *P. aeruginosa* pathogenesis including the ability to form multicellular biofilm (also referred to as aggregates) communities. Biofilm and aggregate formation requires multiple functions, but key factors are exopolysaccharides, which surround the cells and help resist stressors such as bacteriophage and host immune components. The three major polysaccharides produced by *P. aeruginosa* are alginate, PSL and PEL and all have been shown to play a role in biofilm formation *in vitro*. The production of alginate results in a mucoid phenotype, and mucoid strains are commonly isolated from CF lungs. There is variation between *P. aeruginosa* strains as to which types of exopolysaccharide they produce. Biofilm formation is also mediated by adhesins such as lectins, which recognize specific sugars on the surface of neighbouring bacterial cells and also host cells.

QS is a mechanism of cell-to-cell communication in many species of bacteria [5]. In *P. aeruginosa,* a complex QS regulatory cascade involving *N-*acyl homoserine lactone and alkyl-quinolone signal molecules has been linked to the production of a number of toxic exoproducts involved in virulence in a cell-density-dependent manner [5]. QS is thought to regulate around 10 % of the genes in *P. aeruginosa *. QS has been shown to be a social trait in *P. aeruginosa* and has been used extensively to study social evolution theory [6]. Through these studies we now know that cooperative behaviours can be exploited by social cheats and that cheats can impact on virulence during infection [5]. QS is also a target for anti-virulence drugs, which aim to disrupt communication between *P. aeruginosa* cells by either degrading QS signals, or by blocking signal/receptor interactions [5].

*P. aeruginosa* is a prolific producer of exoproducts and secondary metabolites including the antimicrobials/toxins pyocyanin, proteases, exotoxin A and hydrogen cyanide. Many of these are regulated by QS. In addition, it produces two siderophores, pyoverdine and pyochelin, important for scavenging iron in low-iron environments. These secondary metabolites have been shown to be important for *P. aeruginosa* fitness in a number of environments and some have been linked to social behaviours including biofilm formation.

Lipopolysaccharide (LPS) is found in the outer membrane, and is a large molecule consisting of a lipid (lipid A) and a polysaccharide composed of O-antigen and an outer and inner core joined by a covalent bond [7]. LPS increases the negative charge of *P. aeruginosa* cells and it adds structural integrity as well as protecting the membrane from different chemicals. *P. aeruginosa* strains can produce two different types of O-antigen (A and B bands) simultaneously in the same cell [7]. The A band (common antigen) is produced by many *P. aeruginosa* strains and it elicits a weak antibody response. It is a homopolymer of d-rhamnose (d-Rha), commonly around 70 sugars in length. In contrast, the B band (specific antigen), is highly immunogenic, and the nature and number of sugars differs between *P. aeruginosa* strains. B bands can be recognized by O-specific antibodies, which has resulted in the identification of at least 20 major serotypes (O1 to O20). Due to its immunogenic nature, the B band is often lost in strains isolated from chronic infection, resulting in a rough colony phenotype [7]. Interestingly, the switch to a mucoid phenotype from a non-mucoid, often results in the loss of the O-antigen B band.

To date, five protein secretion systems have been identified in *P. aeruginosa* (types I, II, III, V and VI) and each have different functions. The type II system (T2SS) is sometimes referred to as the ‘general secretion pathway’ and promotes outer membrane translocation of large (including some multimeric) exoproteins that are already folded in the periplasm. The type III system (T3SS) controls the injection of toxic proteins, called effectors, directly into the cytosol of host cells. Four key effectors identified in *P. aeruginosa* are ExoU, ExoT, ExoY and ExoS. Once injected, these effectors can result in rapid host cell death. The major phylogenetic groups (I and II) differ in their type III systems. The majority of strains encode either ExoS (group I) or ExoU (group II) with different associated toxins, and this impacts on epithelial cell invasion and/or cytotoxicity. Most recently, the type VI system (T6SS) has been described. This system functions by ‘stabbing’ other *P. aeruginosa* cells resulting in cell death and has been proposed to be used for intra-strain competition. *P. aeruginosa* also produces a range of bacteriocins termed pyocins (S, R and F-types), which kill other sensitive strains of *P. aeruginosa* and are also thought to be used for intra-strain competition.

Pili and flagella are important for swimming, twitching and swarming motility but they also play a role in infection. Pili have been shown to mediate adherence to epithelial cells in culture, and non-piliated *P. aeruginosa* mutants exhibit decreased virulence relative to their parental strains in animal models of pulmonary, intraperitoneal and burned skin infections. Pili-mediated binding to surfaces is thought to be a key first step in biofilm formation [8]. Flagella have been shown to be important in mediating *P. aeruginosa* adhesion to mucin, a glycosylated protein produced by host epithelial tissues and commonly found in CF sputum. Flagella mutants are also often impaired in virulence in acute animal infection models [9].

The resistance-nodulation-cell division (RND) family of efflux pumps are widely found in Gram-negative bacteria [10]. To date, 12 RND efflux pumps have been identified in *P. aeruginosa *. When expressed, RND pumps confer clinically relevant levels of multi-drug resistance and export a wide range of substrates. One of the best characterized RND-efflux pumps in *P. aeruginosa* is the MexAB-OprM system, which may contribute to resistance to high antimicrobial resistance in biofilms. The true biological roles of efflux systems remain to be fully elucidated.

## Open questions

- Are we furthering our understanding of *P. aeruginosa* infection by working with laboratory strains? How accurately do our current infection models capture the physiology of *P. aeruginosa* during human infection?
- Where do the majority of *P. aeruginosa* strains that infect humans come from? Are they transmissible epidemic strains or acquired from the environment?
- How is phenotypic and genotypic heterogeneity generated in *P. aeruginosa* populations during chronic infection? What impact does this have on patient health and treatment? Is the mucoid phenotype during chronic infection (especially in CF lungs) associated with a poorer outcome for patients?
- More emphasis should be placed on ecological and evolutionary questions about *P. aeruginosa* infections. How does spatial structure impact on interactions between *P. aeruginosa* strains and other species of microbes? What is the role of QS and biofilms during infection? Does social cheating between cells occur during infection and what impact does this have on population dynamics, virulence and antibiotic resistance?
- What are the major drivers of AMR and can we develop new therapeutic strategies to counter *P. aeruginosa *?
