# Corrigendum: Microbe Profile: *Pseudomonas aeruginosa*: opportunistic pathogen and lab rat

**DOI:** 10.1099/mic.0.001073

**Published:** 2021-08-12

**Authors:** Stephen P. Diggle, Marvin Whiteley

**Affiliations:** ^1^​ Center for Microbial Dynamics & Infection, School of Biological Sciences, Georgia Institute of Technology, Atlanta, GA 30332, USA

In the published version of this article, there was an error in the second sentence of the section ‘Properties’.

The sentence ‘It is a facultative aerobe that grows via aerobic respiration and anaerobic respiration with nitrate as the terminal electron acceptor.’ should have read ‘It is a facultative anaerobe that grows via aerobic respiration and anaerobic respiration with nitrate as the terminal electron acceptor’.

The authors apologise for any inconvenience caused.

